# Exploratory comparative profiling of 22 macro- and trace elements reveals disrupted elemental homeostasis in colorectal cancer tissues

**DOI:** 10.3389/fonc.2026.1866413

**Published:** 2026-09-01

**Authors:** Juanxian Cheng, Lishan Liang, Linyu Wang, Yan Lu, Cheng Xu, Huabin Gao

**Affiliations:** 1 Department of Pathology, The First Affiliated Hospital, Sun Yat-sen University, Guangzhou, China; 2 Department of Pathology, Jieyang People’s Hospital, Jieyang, China

**Keywords:** colorectal cancer, elemental homeostasis, tissues, trace elements, tumor microenvironment

## Abstract

Colorectal cancer (CRC) is associated with systemic and local alterations in element homeostasis, yet tissue-level data remain limited and inconsistent. This study quantitatively profiled 22 macro- and trace elements in paired tumorous and histopathologically confirmed adjacent non-tumorous colonic tissues from 15 CRC patients using inductively coupled plasma mass spectrometry (ICP-MS). We observed significant accumulation of phosphorus (P), calcium (Ca), magnesium (Mg), iron (Fe), molybdenum (Mo), Rubidium (Rb), strontium (Sr), titanium (Ti), gallium (Ga), niobium (Nb), and zirconium (Zr) in tumor tissues, alongside a significant decrease in cobalt (Co). The intratumoral copper-to-zinc (Cu/Zn) ratio showed a nominal elevation that did not survive false discovery rate (FDR) correction. Critically, the strong positive correlations among macroelements (e.g., P, Ca, Mg) and essential trace elements in human body (e.g., Fe, Zn, Cu, Mo) consistently observed in adjacent non-tumorous tissues were substantially weakened or abolished in tumor tissues, suggesting a systemic collapse of coordinated elemental regulation within the tumor microenvironment. These findings indicate that tumor tissues exhibit accumulation of certain elements, which is associated with the elevated biosynthetic demands of proliferating cells, whereas the broader disruption of elemental networks is correlated with CRC pathogenesis. The widespread disturbance of trace element compositional homeostasis, rather than individual element alterations, may represent a more integrative biomarker for CRC diagnosis and progression.

## 1 Introduction

Colorectal cancer (CRC) remains a major global health burden, ranking as the third most commonly diagnosed malignancy and the second leading cause of cancer-related mortality worldwide (1). Accumulating evidence demonstrates that macro- and trace elements play multifaceted roles in CRC pathogenesis—not only as essential cofactors for antioxidant enzymes (e.g., glutathione peroxidase and superoxide dismutase), but also as critical modulators of redox homeostasis, cell cycle regulation, DNA repair fidelity, and immune function within the tumor microenvironment (2–5). Consistent with this, serum analyses in CRC patients have repeatedly revealed significant dysregulation of key elements—including Zn, Cu, and Se—with the Cu/Zn ratio proposed as a potential circulating biomarker for CRC diagnosis (5–12). Mechanistically, Zn and Se deficiencies are linked to diminished antioxidant defense and increased genomic instability, whereas elevated Cu levels are associated with enhanced angiogenesis, epithelial–mesenchymal transition (EMT), and chronic systemic inflammation (5, 8, 12, 13). Notably, the heterogeneity in serum copper findings is partially explained by analytical methods and data reporting inaccuracies, with sensitivity analyses suggesting that excluding studies with values outside normal ranges reveals significantly higher copper levels in CRC patients (10). Beyond total metal levels, the exchangeable copper (CuExc) pool, representing the labile and toxic fraction of serum copper, has emerged as a promising biomarker; the CuExc-to-zinc ratio demonstrates superior diagnostic accuracy for CRC (AUC = 0.94) compared to traditional Cu/Zn ratios, and correlates with tumor progression and metastasis (13, 14).

In contrast, direct tissue-level comparisons of elemental composition between CRC tumor tissues and matched adjacent non-tumorous colonic tissues remain scarce and yield inconsistent findings. Some studies report that Cu and Zn concentrations in CRC tumor tissues mirror the trends observed in serum, and the Cu/Zn ratio retains potential diagnostic relevance in tumor tissue (3, 4, 15, 16). However, other investigations have found no statistically significant differences in Cu, Zn, Se, or the Cu/Zn ratio between tumor and adjacent non-neoplastic tissues (12, 17). This discrepancy may be attributed to the inherently low abundance of trace elements in biological tissues and the heterogeneity of tumor microenvironment (10). A meta-analysis focusing on tissue copper levels further indicates that pre-analytical procedures—particularly whether samples are processed as “wet” homogenates or “dry” digested tissues—substantially influence measurements, with the former revealing a significant decrease in cytoplasmic copper pools within tumors (15). This cytoplasmic reduction may reflect rapid copper turnover in cancer cells to sustain proliferative and metastatic demands, a process mechanistically linked to EMT via copper-dependent allosteric modulation of kinases such as MEK1/2 and PDK1-AKT pathways (13). However, the potential mechanisms for the discrepancy remain incompletely understood. In addition, existing literature is frequently constrained by limited elemental coverage (typically ≤10 elements), resulting in a lack of consensus regarding both the direction and magnitude of elemental alterations (3, 9, 15–19).

To address these limitations, we performed a comprehensive quantitative analysis of 22 macro- and trace elements (P, Ca, Mg, Fe, Zn, Cu, Mo, Co, Cr, Mn, Ni, V, Li, Pb, Rb, Sr, Ti, Ba, Cs, Ga, Nb, Zr) in paired tumor and histologically confirmed adjacent normal colonic tissues from 15 CRC patients through the method of inductively-coupled plasma mass-spectrometry (ICP-MS). By integrating our findings with existing literature and applying robust statistical modeling, we define a refined, tissue-based elemental signature of the CRC tumor microenvironment and systematically evaluate its associations with clinicopathological parameters and molecular pathways central to colorectal carcinogenesis.

## 2 Materials and methods

### 2.1 Patients and samples

**Figure 1:**
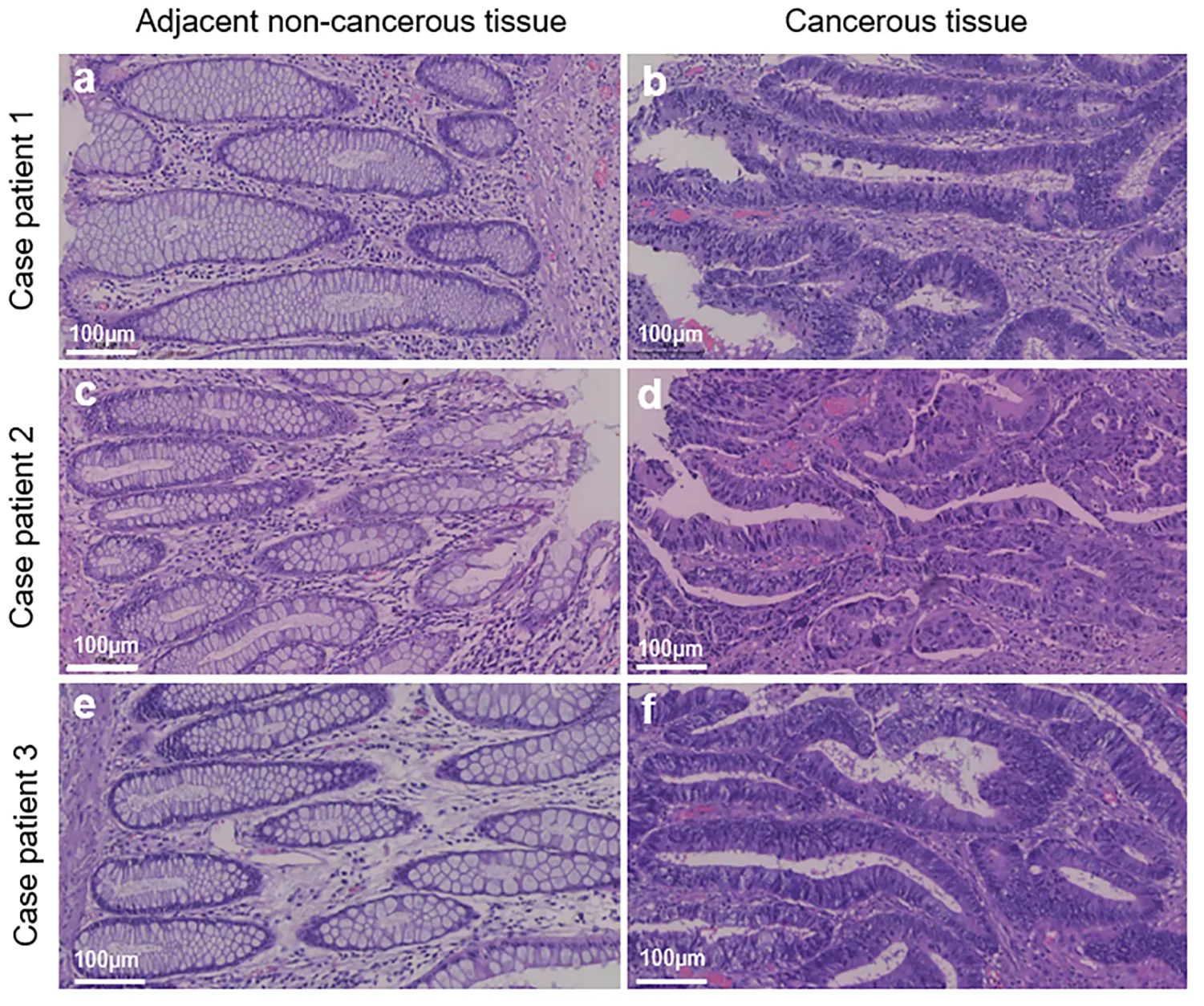
Hematoxylin–eosin (H&E) staining of cancerous and adjacent non-cancerous tissues from three representative colorectal cancer patients.

Paired tumorous and adjacent non-tumorous tissues were obtained from 15 patients diagnosed with invasive colorectal adenocarcinoma at the Department of Pathology, First Affiliated Hospital of Sun Yat-sen University (Guangzhou, China), prior to any neoadjuvant chemotherapy or radiotherapy. A total of 30 tissue specimens were collected—15 tumor samples and 15 matched adjacent non-neoplastic tissues. Hematoxylin–eosin (H&E) staining confirmed the absence of dysplasia or malignancy in all adjacent normal tissues, whereas tumor specimens exhibited characteristic histopathological features, including marked cellular hypercellularity, loss of glandular architecture, and dense infiltration of atypical epithelial cells within the colonic mucosa and submucosa (**Figure 1**).

Basic characteristics of the 15 CRC patients are summarized in **Table 1**. The cohort comprised 13 males and 2 females, with the majority of patients (73%) aged ≥60 years. All participants had no documented history of comorbidities such as hypertension or diabetes mellitus. Tumors were predominantly located in the rectum (60%), with the maximum tumor diameter ranging from 2 to 7cm (median, 4.8cm). Lymph node metastasis was observed in 2 patients (13%). Tumor stage was classified as I (localized tumor), II (local spread), III (extensive regional spread), or IV (distant metastasis), following the criteria of Lavilla et al. (3). In our cohort, the majority of patients presented with stage II disease (60%), followed by stage I (27%) and stage III (13%); no stage IV cases were included (**Table 1**). All tissue sections were reviewed by two independent pathologists to estimate tumor cellularity (>70%), necrosis extent (<10%), hemorrhage, stromal content, and inflammatory infiltration. Samples with extensive necrosis or hemorrhage were excluded from analysis. All the tissue samples were snap-frozen in liquid nitrogen and stored at −80 °C prior to lyophilization. Lyophilization was performed under vacuum (≤0.1 mbar) at −80 °C for 72 h until constant mass was achieved. This study was approved by the Ethics Committee for Clinical Research and Animal Trials of the First Affiliated Hospital of Sun Yat-sen University (Approval No. [2023]300).

**Table 1:** Basic characteristics of the 15 CRC patients in this study.

Variables	CRC patients (n=15)	
	n	%
Gender		
Male	13	87
Female	2	13
Age		
50–60 years	4	27
≥ 60 years	11	73
Tumor location		
Rectum	9	60
Colon	6	40
Lymph node metastasis		
Positive	2	13
Negative	13	87
Tumor infiltration		
Positive	15	100
Negative	0	0
Tumor stage		
I	4	27
II	9	60
III	2	13
IV	0	0

### 2.2 Elements analysis

Wet digestion of the lyophilized tissue samples was performed following a modified aqua regia procedure. All digestion vessels and PFA beakers were acid-cleaned before use. Approximately 50 mg of lyophilized tissue was accurately weighed into Teflon digestion bombs. A mixture of 2 mL of 15.34 N HNO_3_ and 0.5 mL of 23 N HF was added, and the sealed bombs were placed in stainless-steel jackets and heated at 190 °C for 72 h. After cooling, the digest was transferred into PFA beakers in a clean fume hood; the bombs were rinsed twice with 1 mL of 1 N HNO_3_, and the rinsates were combined with the digest. The combined solution (~4.5 mL) was evaporated to dryness at 110 °C. The residue was treated with 4 mL of aqua regia at 120 °C overnight, then evaporated to dryness. Subsequently, 2 mL of 7 N HNO_3_ and then 2 mL of 2 N HNO_3_ were added sequentially, each followed by evaporation at 110 °C. Finally, 3 mL of 2 N HNO_3_ was added, and the capped beakers were heated at 120 °C overnight to dissolve the residue. After cooling, the solutions were weighed and adjusted gravimetrically for ICP-MS analysis (20, 21).

Elemental concentrations were quantified using a Thermo Fisher Scientific iCAP RQ inductively coupled plasma mass spectrometer (ICP-MS) at Guizhou Tongwei Testing Technology Co., Ltd. (China). Calibration standards and sample solutions were prepared gravimetrically using a high-precision analytical balance (± 0.01 mg). Internal standardization was performed with indium (In) and rhenium (Re), both added online at fixed concentrations (1 ng/mL each) to correct for instrumental drift and matrix effects. Calibration was established using certified reference materials (CRMs): basalt BHVO-2, tungsten ore W-2a, and a multi-element Mo standard solution (NIST SRM 3134). Method precision, assessed via replicate digestions (n = 6) of a representative tissue sample, yielded relative standard deviations (RSDs) < 5% for all reported elements. Molybdenum quantification was further validated against the NIST Mo standard (SRM 3134) via separate single-element calibration. The concentrations of the 22 elements measured in all 30 tissue specimens (15 tumor samples and 15 matched adjacent non-tumor tissues) are presented in the **Supplementary Table 1** of the **Supplementary Materials**.

### 2.3 Statistical analyses

Elemental concentrations in tissue samples are presented as mean ± standard deviation, median, and range. Differences in elemental composition between colorectal cancer tumorous tissues and matched adjacent non-tumorous tissues were evaluated using the paired t-test when the distribution of paired differences approximated normality (as confirmed by Shapiro–Wilk tests); otherwise, the Wilcoxon signed-rank test was employed. Statistical significance was defined as *P* < 0.05 (uncorrected). Given the exploratory nature of this study and the simultaneous testing of 22 individual elements plus the Cu/Zn ratio (totaling 23 hypotheses), the Benjamini–Hochberg procedure was applied to control the false discovery rate (FDR), with statistical significance defined as an FDR-adjusted q-value < 0.05 (the calculated results were shown in the **Supplementary Table 2** of **Supplementary Materials**). Due to the limited sample size (n = 15 matched pairs) and the non-normal distribution observed for several elemental concentrations (per Shapiro–Wilk tests), inter-element correlations were evaluated using Spearman’s rank correlation coefficient. All statistical analyses were conducted using OriginPro 2021 (OriginLab Corporation, Northampton, MA, USA).

## 3 Results

A total of 22 elements, comprising 3 major elements (P, Ca, Mg) and 19 trace elements (Fe, Zn, Cu, Mo, Co, Cr, Mn, Ni, V, Li, Pb, Rb, Sr, Ti, Ba, Cs, Ga, Nb, Zr), were quantified in both tumorous and adjacent non-tumorous tissues from patients with colorectal cancer (**Table 2**). Based on their mean concentrations, the 22 measured elements were stratified into two distinct categories: seven elements (P, Ca, Mg, Fe, Zn, Cu, and Rb) exhibited mean concentrations >1 μg/g, whereas the remaining 15 elements were detected at sub-μg/g levels (ranging from ng/g to ~1 μg/g).

**Table 2:** Elemental concentrations in CRC tumorous and adjacent non-tumorous tissues.

Element/ parameter	Adjacent non-tumorous tissue (n=15)			Tumorous tissue (n=15)			*P* value (uncorrected)	q value (FDR-adjusted)
	Mean ± SD	Median	Range	Mean ± SD	Median	Range		
P (μg/g)	4191.26 ± 1996.69	3918.14	1930-7960	7564.23 ± 3017.91	8460.00	2627.32-11300	0.001	0.004**
Ca (μg/g)	464.48 ± 235.27	416.00	162-1010	646.05 ± 246.39	621.32	307-1190	0.025	0.048*
Mg (μg/g)	419.73 ± 232.11	308.82	145-853	688.25 ± 210.10	707.00	354-961	0.001	0.004**
Fe (μg/g)	60.22 ± 22.35	60.15	17.3-98.3	141.73 ± 81.48	150.00	29.1-287	0.001	0.004**
Zn (μg/g)	47.17 ± 17.53	48.57	19.9-76.73	40.31 ± 11.38	41.90	19.9-59.6	0.194	0.262
Cu (μg/g)	5.52 ± 2.88	6.23	1.46-10.1	6.13 ± 2.22	6.39	2-10	0.507	0.530
Mo (ng/g)	30.79 ± 10.75	31.38	17.4-48.6	45.82 ± 13.63	47.66	26.6-80.4	0.003	0.010**
Co (ng/g)	17.33 ± 6.16	15.40	7.78-28.75	10.43 ± 5.71	7.67	3.68-21.8	0.007	0.018*
Cr (ng/g)	199.87 ± 49.49	185.00	152.66-300	183.11 ± 65.13	174.00	60.09-282	0.256	0.310
Mn (ng/g)	805.57 ± 450.13	675.53	258-1700	1032.23 ± 508.64	943.00	493-2010	0.256	0.310
Ni (ng/g)	181.75 ± 122.81	148.00	51.9-486	223.04 ± 155.81	218.56	25.15-501	0.336	0.368
V (ng/g)	21.07 ± 14.57	16.20	9.93-54.8	32.48 ± 18.41	29.20	10.7-70.5	0.057	0.094
Li (ng/g)	15.23 ± 7.84	12.30	6.79-31.04	19.37 ± 10.61	15.60	6.97-38.3	0.293	0.337
Pb (ng/g)	56.88 ± 42.63	49.60	19-195	55.66 ± 45.99	38.30	10.6-199	0.944	0.944
Rb (ng/g)	4531.60 ± 3841.50	3290.00	696-10698	6163.26 ± 3578.39	4550.00	2720-14600	0.025	0.048*
Sr (ng/g)	513.79 ± 281.70	592.91	132-940	916.82 ± 670.88	859.00	160.36-2370	0.011	0.025*
Ti (ng/g)	340.13 ± 112.55	301.27	190-618	455.89 ± 76.65	455.00	323-566.98	0.001	0.004**
Ba (ng/g)	159.14 ± 75.84	164.24	44-301	219.09 ± 118.15	227.00	34-412	0.08	0.123
Cs (ng/g)	22.16 ± 16.84	22.40	3.47-46.9	26.49 ± 16.00	18.10	2.1-61.5	0.164	0.236
Ga (ng/g)	11.34 ± 5.41	9.52	4.72-23.4	20.95 ± 7.99	22.88	5.15-31.1	0.001	0.004**
Nb (ng/g)	8.08 ± 2.41	8.74	3.64-11.8	17.99 ± 10.90	15.90	3.17-41.6	0.001	0.004**
Zr (ng/g)	7.34 ± 3.50	6.43	2.55-16	14.47 ± 8.08	14.10	3.31-33.9	0.006	0.017*
Cu/Zn ratio	0.11 ± 0.04	0.10	0.07-0.22	0.16 ± 0.07	0.14	0.08-0.31	0.044	0.078

CRC, colorectal cancer; SD, standard deviation; P, phosphorus; Ca, calcium; Mg, magnesium; Fe, iron; Zn, zinc; Cu, Copper; Mo, molybdenum; Co, Cobalt; Cr, chromium; Mn, manganese; Ni, nickel; V, vanadium; Li, lithium; Pb, plumbum; Rb, rubidium; Sr, strontium; Ti, titanium; Ba, barium; Cs, caesium; Ga, gallium; Nb, niobium; Zr, zirconium; Concentrations are based on wet tissue weight after lyophilization and digestion; *Indicates the statistically significant difference, *q<0.05; **q<0.01; ***q<0.001 (FDR-adjusted by Benjamini–Hochberg method).

In tumor tissues versus adjacent normal tissues, 12 elements showed statistically significant concentration differences after FDR correction (q < 0.05). Among macroelements, P, Ca, and Mg were all significantly elevated in tumor tissues. Among the six essential trace elements quantified (Fe, Zn, Cu, Mo, Co, Cr), Fe and Mo were significantly enriched in tumor tissue, while Co was significantly depleted; Zn and Cu showed no significant intergroup differences, and the Cu/Zn ratio showed a nominal elevation but did not survive false discovery rate (FDR) correction. Among the remaining trace elements, Rb, Sr, Ti, Ga, Nb, and Zr were significantly accumulated in tumors, whereas Mn, Ni, V, Li, Pb, Ba, and Cs exhibited no statistically significant variation between tumor and adjacent normal tissues. Comprehensive quantitative data, including means, standard deviations, medians, ranges, and Benjamini–Hochberg adjusted q-values, are presented in **Table 2**.

## 4 Discussion

### 4.1 Elemental concentration alternations between tumorous and adjacent non-neoplastic tissues from CRC patients

A total of 12 elements exhibited statistically significant differences between tumorous and adjacent non-tumorous tissues after FDR correction (q < 0.05) (**Table 2**; **Supplementary Figure 1** in the **Supplementary Materials**). These include three macroelements (P, Ca, and Mg), two essential trace elements (Fe and Mo, both elevated in tumors) with Co being significantly decreased, and six other trace elements (Rb, Sr, Ti, Ga, Nb, and Zr) all showing significant accumulation in tumor tissues. Notably, the Cu/Zn ratio was elevated in tumor tissues at the nominal level (uncorrected *P* = 0.044), but this difference did not withstand FDR correction (q = 0.078).

#### 4.1.1 Elements showing significant accumulation in tumor tissues

A consistent pattern emerged for the macroelements (P, Ca, and Mg), all of which were significantly enriched in tumor tissues (q < 0.05) (**Table 2**). This enrichment is likely linked to the heightened metabolic demands of proliferating cancer cells. P serves as a structural and functional cornerstone of nucleic acids and ATP, directly supporting the biosynthetic and energetic requirements of rapidly dividing tumor cells (22, 23); moreover, the elevated glycolytic flux characteristic of cancer metabolism necessitates abundant phosphate for protein phosphorylation cascades and *de novo* nucleotide synthesis (24). Ca signaling is pathologically remodeled in malignancy to promote proliferation, invasion, and immune evasion (25–27); notably, abnormal Ca accumulation is a well-documented feature of necrotic tumor regions, particularly in advanced-stage colorectal cancer, where disrupted Ca homeostasis, driven by dysregulated ion channels and pumps (e.g., TRPC channels, SERCA, PMCA), may lead to intracellular overload (28, 29). Mg acts as an essential cofactor for over 600 enzymatic reactions, including those governing glycolysis, oxidative phosphorylation, DNA replication, and cell cycle progression (30, 31); it also promotes angiogenesis via modulation of vascular endothelial growth factor (VEGF) expression and endothelial cell motility (32). Collectively, our observations align with prior reports in colorectal cancer (3, 4, 12, 19), reinforcing macroelement enrichment as a defining feature of the CRC metabolic phenotype.

Among essential trace elements, Fe and Mo were significantly elevated in tumor tissues (q < 0.05) (**Table 2**). Fe accumulation reflects the well-characterized “iron addiction” of malignant cells (33). In CRC, dysregulated iron homeostasis, marked by upregulated transferrin receptor 1 (TFR1) and downregulated ferroproteins, results in intracellular iron retention, fueling DNA synthesis, ROS-mediated pro-tumorigenic signaling, angiogenesis, and loss of epithelial integrity (34–38). Our Fe results align with those of Lavilla et al. (3), although other studies have reported no significant difference or even reduced Fe concentrations in CRC tissues (4, 12, 17, 18). These discrepancies are likely associated with cohort heterogeneity, tumor stage, or analytical methodology. Mo functions as an obligate cofactor for four evolutionarily conserved enzymes, including sulfite oxidase, xanthine oxidase, aldehyde oxidase, and the mitochondrial amidoxime-reducing component (mARC), that collectively regulate sulfur amino acid metabolism, purine catabolism, and detoxification of xenobiotics and endogenous metabolites (39). Although Mo has been infrequently investigated in CRC, our observation of significant Mo enrichment contrasts with reports of unchanged or reduced levels (3, 12, 17) and may indicate heightened activity of molybdenum-dependent enzymes (particularly mARC) in supporting redox balance and metabolic adaptation within the tumor microenvironment (40).

For the remaining trace elements, Rb, Sr, Ti, Ga, Nb, and Zr were all significantly accumulated in tumor tissues (q < 0.05). Sr depletion has been previously reported in early-stage CRC (16); however, our data reveal the opposite trend. Data on the concentrations of Rb, Ti, Ga, Nb, and Zr in CRC tissues are scarce in the existing literature, our findings demonstrate significant accumulation of these elements in colorectal tumors. The biological significance of these elements in CRC pathogenesis warrants further investigation.

#### 4.1.2 Elements with decreased or non-significant changes in tumor tissues

In contrast to the broad pattern of elemental enrichment, Co was significantly depleted in tumor tissues (q < 0.05). As the central metal ion of vitamin B_12_, Co is indispensable for methionine synthase–mediated one-carbon metabolism, DNA synthesis, and erythropoiesis (41). Paradoxically, cobalt ions can stabilize hypoxia-inducible factor-1α (HIF-1α) under normoxic conditions, inducing a pseudo-hypoxic transcriptional program that enhances angiogenesis and metastatic potential (42). Our observation of reduced Co concentration aligns with Zhang et al. (17), whereas other independent studies found no significant intergroup difference (3, 4, 12). This depletion may reflect reduced vitamin B_12_-dependent metabolic activity or altered cobalt utilization within the tumor microenvironment.

Several elements showed no statistically significant differences after FDR correction (q ≥ 0.05) (**Table 2**), including Zn, Cu, Cr, Mn, Ni, Li, Pb, Ba, and Cs. Among these, Zn and Cu warrant focused interpretation due to their well-established roles in redox regulation, DNA metabolism, and tumor progression. Cu serves as an essential cofactor for key enzymes governing redox homeostasis and angiogenesis (5). Although multiple studies have reported elevated intratumoral Cu concentrations that correlate with advanced stage and poor prognosis (16, 43), our results, consistent with Zhang et al. (17), show only a non-significant upward trend (q = 0.530), likely reflecting cohort-specific heterogeneity in tumor grade, stromal infiltration, or analytical sensitivity. Zn functions as a structural and catalytic cofactor for over 300 metalloenzymes, including DNA polymerases, thymidine kinase, and superoxide dismutase, and is indispensable for genomic stability, cell cycle control, and epithelial barrier integrity (8). Our quantitative analysis revealed slightly decreased Zn levels in CRC tissues, which is well corroborated by Juloski et al. (4) and Zhang et al. (17). However, discrepancies persist across different studies. Lavilla et al. (3) and Skalny et al. (12) reported no significant difference, while Sohrabi et al. (18) observed significantly higher Zn in tumors. Crucially, although neither Zn nor Cu alone reached statistical significance after FDR correction, the intratumoral Cu/Zn molar ratio was elevated at the nominal level (uncorrected *P* = 0.044; q = 0.078), falling just short of the corrected threshold. This observation aligns with an increasing body of evidence indicating that elemental ratios (not absolute concentrations) may better capture biologically meaningful dysregulation. The serum Cu/Zn ratio has been validated as a clinically useful biomarker for CRC detection and risk stratification (2, 6, 7, 12, 44–46), and independent tissue-based studies (including ours) consistently report elevated intratumoral Cu/Zn ratios relative to adjacent non-tumorous tissues (3, 4, 16, 43). This supports the concept that the balance between different elements, rather than individual concentrations, may be more biologically relevant. Beyond static concentration measurements, emerging evidence highlights the functional relevance of copper dyshomeostasis in CRC through cuproptosis—a copper-dependent form of regulated cell death (5, 11, 47). The exchangeable copper pool (CuExc) and its ratio to Zn (CuExc: Zn) have demonstrated exceptional diagnostic performance, achieving an AUC of 0.94 for discriminating CRC patients from healthy controls (13). Furthermore, copper imbalance has been mechanistically linked to epithelial–mesenchymal transition (EMT), a key driver of metastasis, and mutant KRAS has been shown to promote copper uptake via macropinocytosis to sustain tumor growth (13).

For the remaining non-significant elements, the absence of intergroup differences for Mn, Ni, and Pb is consistent with multiple previous reports (3, 17, 18), suggesting these elements are unlikely to be primary drivers of CRC-associated elemental dysregulation at the tissue level, despite their established roles as enzymatic cofactors (Mn, Ni) or environmental carcinogens (Pb) (48–50). Mn concentration levels have been reported to be elevated in CRC tissues in some studies, attributed to upregulated expression of manganese-dependent superoxide dismutase (Mn-SOD) as an adaptive antioxidant response (3, 16), but our data did not achieve statistical significance after FDR correction (q = 0.310). V and Li remain comparatively understudied in CRC pathobiology. Although systemic V levels have been reported to be correlated with clinical disease stage (12), our tissue-level data showed no significant intergroup difference (uncorrected *P* = 0.057; q = 0.094). Pb, a well-established human carcinogen, exhibits highly variable tissue distribution across studies (3, 18), and our analysis revealed no statistically significant intergroup difference (q = 0.944).

#### 4.1.3 Summary of elemental alteration patterns

Collectively, the concentration alterations observed in this study reveal a non-uniform pattern. Macroelements (P, Ca, Mg) and a subset of trace elements (Fe, Mo, Rb, Sr, Ti, Ga, Nb, Zr) are consistently enriched in CRC tumor tissues, likely reflecting the elevated biosynthetic and metabolic demands of proliferating cancer cells. In contrast, essential trace elements such as Zn and Cu show no individual concentration changes, yet their ratio (Cu/Zn) is altered at the nominal level, suggesting that elemental homeostasis, rather than absolute abundance, is disrupted within the tumor microenvironment. The distinct accumulation profiles of rarely studied elements (Rb, Ti, Ga, Nb, and Zr) highlight the need for further mechanistic studies to elucidate their potential roles in colorectal carcinogenesis. Overall, these heterogeneous alterations, spanning significant enrichment, significant depletion, and non-significant changes, collectively support the concept of a nonlinear and systemic disruption of trace element homeostasis in the CRC tumor microenvironment.

### 4.2 Correlation analysis of elements in tumorous and adjacent non-neoplastic tissues from CRC patients

**Figure 2:**
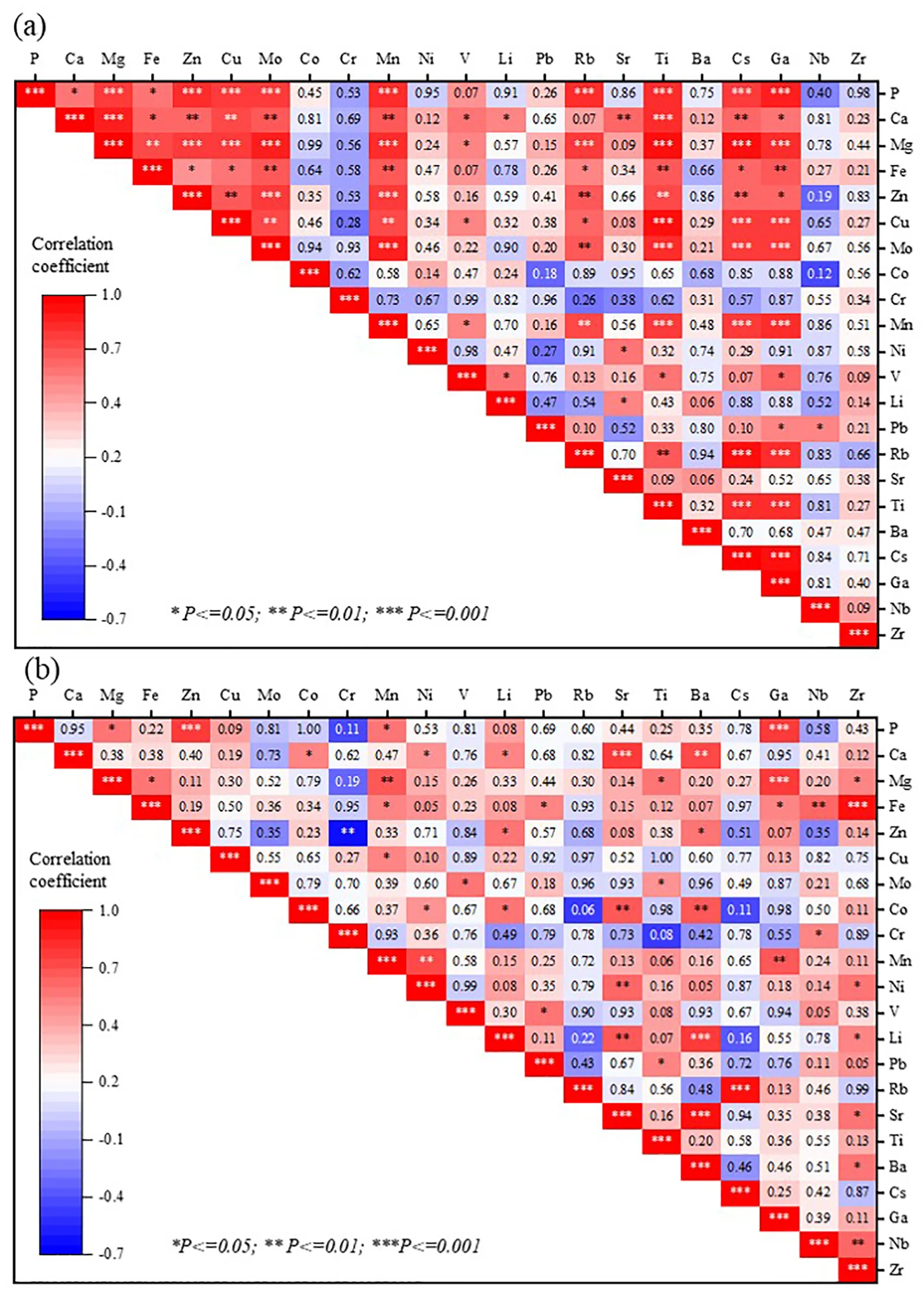
Spearman correlation among 3 macroelements (P, Ca, and Mg), 6 essential trace elements (Fe, Zn, Cu, Mo, Co, and Cr) and other 13 trace elements in **(a)** adjacent non-neoplastic and **(b)** tumorous tissues from CRC patients. *P≤0.05; **P≤0.01; ***P≤0.001.

Correlation analysis of the 22 major and trace elements in CRC cancerous and adjacent non-tumorous tissues is shown in **Figure 2**. In adjacent non-tumorous tissues, the major elements P, Ca, and Mg show a good positive correlation (**Figure 2a**). Similarly, the essential trace elements Fe, Zn, Cu, and Mo also exhibit a good positive correlation, while the essential trace elements Co and Cr show negative correlations with the other 21 elements. Additionally, the contents of Mn, V, Rb, Ti, Cs, and Ga are positively correlated with those of P, Ca, Mg, Fe, Zn, Cu, and Mo, respectively (**Figure 2a**). However, no obvious regular correlation is observed among the contents of Ni, V, Li, Pb, Rb, Sr, Ti, Ba, Cs, Ga, Nb, and Zr.

In contrast, in CRC tumor tissues, the positive correlations among P, Ca, Mg, Fe, Zn, Cu, and Mo are significantly weakened, and the correlation between Mo and the other six elements changes from positive to negative (**Figure 2b**). Similarly, the negative correlations between Co and Cr and the other 21 elements are also significantly weakened, and some elements have changed to positive correlations. In CRC tumor tissues, the contents of Mn, V, Rb, Ti, Cs, and Ga are negatively correlated with those of P, Ca, Mg, Fe, Zn, Cu, and Mo, respectively. However, the contents of Ni, V, Li, Pb, Rb, Sr, Ti, Ba, Cs, Ga, Nb, and Zr show no obvious regular correlation, just as in the adjacent non-tumorous tissues.

This indicates that in the microenvironment of CRC tumor tissues, the originally normal dynamic balance of major and trace element contents has been disrupted, intensifying oxidative stress and the destruction of key signaling pathways, providing more favorable microenvironmental conditions for the rapid proliferation and metastasis of tumor cells.

### 4.3 Implications of elemental dysregulation in the colorectal cancer microenvironment

A growing body of evidence indicates that colorectal cancer is associated with significant dysregulation of macroelements and several essential trace elements—both systemically (in serum) and locally (within the tumor microenvironment)—suggesting that altered elemental homeostasis is mechanistically linked to tumor initiation, progression, and metabolic reprogramming.

In serum, the most consistently reported alterations include significantly reduced concentrations of Se and Zn in CRC patients relative to healthy controls (2, 7, 12). These deficiencies impair antioxidant defense systems—particularly selenoprotein-dependent pathways and Cu/Zn-superoxide dismutase (Cu/Zn-SOD)—thereby exacerbating oxidative stress, promoting DNA damage, and facilitating carcinogenesis (3). Conversely, serum Cu levels and the Cu/Zn ratio are frequently elevated in CRC, reflecting a systemic pro-inflammatory and pro-angiogenic milieu that supports tumor growth, invasion, and metastatic dissemination (6, 12, 44).

Within tumor tissues, a distinct elemental signature emerges. CRC lesions consistently exhibit elevated concentrations of macroelements—including P, calcium Ca, K, S, and Mg—compared with matched adjacent non-neoplastic tissues (3). Several essential trace elements—including Fe, Cu, Se, and Mn—function as catalytic cofactors for redox-active enzymes and signaling molecules involved in proliferation, epithelial–mesenchymal transition, and extracellular matrix remodeling (4, 5, 16, 17). However, their tissue-level abundance shows high heterogeneity between different studies, depending on cohort characteristics, analytical methodology, and tumor stage, individual elements may be increased, decreased, or unchanged relative to adjacent non-tumorous tissues. Despite this variability, the collective disruption of trace element homeostasis within the tumor microenvironment is robustly documented, which is increasingly recognized as a functional driver of malignant transformation and progression.

The remarkable contrast between systemic depletion (e.g., Se, Zn in serum) and localized accumulation or redistribution of elements (e.g., P, Ca, Fe, Cu in tumor tissue) points to active, tumor-driven elemental reallocation. Emerging models propose that CRC tumors function as metabolic “sinks,” preferentially sequestering essential micronutrients from circulation to sustain heightened biosynthetic demands, redox adaptation, and resistance to microenvironmental stress (4, 12). Consequently, integrated analysis of serum and tissue elemental profiles offers complementary, spatially resolved insights into the systemic metabolic adaptations and local biochemical dependencies of colorectal cancer.

### 4.4 Study limitations and strengths

Several limitations of this study should be acknowledged. First, the relatively small sample size (n = 15 paired tissues) limits statistical power and precludes robust subgroup analyses based on tumor stage, grade, or molecular subtypes. Consequently, our findings are preliminary and hypothesis-generating, requiring validation in larger cohorts. Second, selenium (Se), a trace element with well-documented antioxidant functions in colorectal cancer (51), was not provided in this study due to the difficulty in achieving precise quantification, despite meta-analyses showing altered Se levels in CRC tissues (9). Third, variations in tumor purity, stromal content, and inflammatory infiltration were not quantitatively evaluated and may partially confound the observed differences. Fourth, the bulk digestion approach provides average elemental concentrations across whole tissues but cannot resolve spatial heterogeneity at the cellular or subcellular level, masking distinct elemental profiles of cancer cells, immune cells, fibroblasts, and extracellular matrix. Future *in situ* microanalytical techniques, such as laser ablation–ICP–MS or synchrotron radiation X-ray fluorescence, could map elemental distributions at high resolution and correlate them with specific histological features.

Despite these limitations, this study offers several notable strengths, including the comprehensive profiling of 22 elements (broader than most previous reports), the paired tissue design minimizing inter-individual variation, and rigorous FDR correction for multiple comparisons. In addition, the significant accumulation of rarely investigated elements (Rb, Ti, Ga, Nb, and Zr) in CRC tissues were reported for the first time in this study, providing a foundation for future mechanistic investigations. Most importantly, our findings point to the possibility that disruption of the overall trace element compositional homeostasis, rather than individual element alterations, may offer a more integrative biomarker for CRC diagnosis and progression.

## 5 Conclusions

In this study, the concentration of 22 elements, including three macroelements (P, Ca, and Mg), six essential trace elements (Fe, Cu, Zn, Mo, Co, and Cr), and 13 other trace elements, in paired tissues and adjacent non-tumorous tissues from CRC patients were quantitatively analyzed. Coupled with results from existing literature, we found that macroelements (e.g., P, Ca, Mg) consistently exhibited significant accumulation in cancerous tissues, which is associated with rapid tumor cell proliferation. In contrast, trace element levels varied across different studies, probably due to their lower baseline concentrations, which render accumulation effects less pronounced. In adjacent normal tissues, strong positive correlations were observed among macroelements (P, Ca, Mg) and essential trace elements (Fe, Zn, Cu, Mo), while Co and Cr showed negative correlations with these elements. Notably, these regular correlation patterns were absent in CRC tumor tissues, indicating severe disruption of elemental homeostasis within the tumor microenvironment. We propose that rapid tumor proliferation may drive localized accumulation of macroelements and relatively abundant trace elements, while concurrently destabilizing trace element homeostasis, and may be associated with tumor progression. Compared to individual element levels or ratios (e.g., Cu/Zn), the widespread disruption of the trace element compositional homeostasis may serve as a more integrative clinical biomarker for colorectal cancer diagnosis.

## Data Availability

The original contributions presented in the study are included in the article/**Supplementary Material**. Further inquiries can be directed to the corresponding authors.
