# Supplementary material for: Exploratory comparative profiling of 22 macro- and trace elements reveals disrupted elemental homeostasis in colorectal cancer tissues
Source: Front Oncol. 2026 Sep 1;16:1866413. doi: 10.3389/fonc.2026.1866413 (PMC13574630; doi:10.3389/fonc.2026.1866413)
Supplement: Supplementary file 1 [file SupplementaryFile1.docx]

Supplementary materials for

“Exploratory comparative profiling of 22 macro- and trace elements reveals disrupted elemental homeostasis in colorectal cancer tissues”

Juanxian Cheng^a, *^, Lishan Liang^a^, Linyu Wang^a^, Yan Lu^b^, Cheng Xu^a^, Huabin Gao^a, *^

^a^ Department of Pathology, The First Affiliated Hospital, Sun Yat-sen University, Guangzhou, China

^b^ Department of Pathology, Jieyang People's Hospital, Jieyang, China

*Corresponding author: Juanxian Cheng (juanxian_cheng@126.com) and Huabin Gao (gaohb8@mail.sysu.edu.cn)

Current address of corresponding author: First Affiliated Hospital of Sun Yat-sen University, No. 8 Hengshun Road, Nansha District, Guangzhou 511466, P.R. China..

Contents of this file:

Figures S1

Table S1 to S2

Figue S1. Comparative analysis of elemental concentrations of tumorous and adjacent non-tumorous tissues from colorectal cancer patients. Data expressed as median (line) and the respective interquartile range (box) and non-outlier range (whiskers). Concentrations are based on wet tissue weight after lyophilization and digestion. Group differences with q values < 0.05 are considered significant. * q<0.05; ** q<0.01; *** q<0.001 (FDR-adjusted by Benjamini–Hochberg method)

Table S1. The concentrations of 22 elements within the 30 tissue specimens from 15 CRC patients in this study

| Patient No. | Sample ID | P | Ca | Mg | Fe | Zn | Cu | Mo | Co | Cr | Mn | Ni | V | Li | Pb | Rb | Sr | Ti | Ba | Cs | Ga | Nb | Zr |
| --- | --- | --- | --- | --- | --- | --- | --- | --- | --- | --- | --- | --- | --- | --- | --- | --- | --- | --- | --- | --- | --- | --- | --- |
|  |  | μg/g | μg/g | μg/g | μg/g | μg/g | μg/g | ng/g | ng/g | ng/g | ng/g | ng/g | ng/g | ng/g | ng/g | ng/g | ng/g | ng/g | ng/g | ng/g | ng/g | ng/g | ng/g |
| 1 | Non-tumorous-01 | 3918.14 | 368.37 | 263.18 | 49.96 | 48.57 | 8.56 | 23.94 | 26.78 | 165.71 | 675.53 | 173.57 | 18.23 | 20.07 | 38.41 | 1523.02 | 614.99 | 347.39 | 206.44 | 8.45 | 9.52 | 5.56 | 11.08 |
|  | Tumorous-01 | 11243.64 | 621.32 | 722.47 | 64.39 | 54.13 | 6.88 | 43.38 | 7.44 | 111.26 | 1060.90 | 218.56 | 31.88 | 36.16 | 97.20 | 3760.33 | 1093.13 | 566.98 | 283.26 | 17.78 | 20.13 | 8.08 | 12.80 |
| 2 | Non-tumorous-02 | 6085.55 | 449.78 | 588.01 | 60.28 | 76.73 | 6.23 | 31.38 | 19.72 | 152.66 | 1307.21 | 76.02 | 29.84 | 14.05 | 49.64 | 10697.89 | 254.96 | 301.27 | 71.60 | 37.75 | 13.90 | 4.95 | 4.26 |
|  | Tumorous-02 | 2627.32 | 336.73 | 566.06 | 50.21 | 26.26 | 2.00 | 52.60 | 4.44 | 60.09 | 512.49 | 25.15 | 20.91 | 6.97 | 36.94 | 3591.55 | 160.36 | 501.44 | 53.25 | 18.10 | 5.15 | 3.17 | 3.31 |
| 3 | Non-tumorous-03 | 6055.16 | 455.02 | 308.82 | 60.16 | 68.00 | 6.69 | 33.58 | 28.75 | 285.62 | 799.78 | 147.16 | 51.84 | 31.04 | 21.62 | 1212.03 | 592.91 | 401.31 | 164.24 | 7.40 | 13.35 | 3.64 | 8.51 |
|  | Tumorous-03 | 11022.43 | 649.66 | 681.24 | 68.80 | 54.20 | 7.58 | 47.66 | 15.87 | 165.34 | 573.02 | 58.48 | 44.01 | 26.85 | 33.93 | 3153.06 | 625.81 | 376.00 | 205.11 | 17.03 | 22.88 | 4.78 | 6.17 |
| 4 | Non-tumorous-04 | 2070.00 | 651.05 | 291.00 | 46.40 | 38.00 | 2.90 | 23.30 | 15.40 | 238.00 | 535.00 | 284.00 | 11.70 | 30.30 | 34.10 | 696.00 | 600.00 | 270.00 | 301.00 | 4.68 | 5.56 | 9.06 | 6.85 |
|  | Tumorous-04 | 2990.00 | 1190.00 | 369.00 | 92.80 | 43.90 | 3.39 | 26.60 | 21.80 | 182.00 | 493.00 | 252.00 | 10.70 | 31.00 | 29.50 | 3184.00 | 2370.00 | 383.00 | 412.00 | 2.10 | 7.03 | 13.90 | 15.20 |
| 5 | Non-tumorous-05 | 3520.00 | 447.00 | 395.00 | 70.60 | 53.40 | 5.04 | 35.30 | 11.80 | 188.00 | 533.00 | 320.00 | 10.20 | 10.50 | 33.90 | 3290.00 | 739.00 | 301.00 | 188.00 | 22.40 | 7.24 | 8.30 | 4.93 |
|  | Tumorous-05 | 8460.00 | 672.00 | 915.00 | 200.00 | 44.00 | 5.35 | 49.80 | 7.67 | 130.00 | 686.00 | 77.50 | 31.50 | 14.10 | 44.70 | 7830.00 | 1320.00 | 455.00 | 322.00 | 40.50 | 25.20 | 26.00 | 20.00 |
| 6 | Non-tumorous-06 | 1930.00 | 364.00 | 205.00 | 50.30 | 22.50 | 2.57 | 20.20 | 7.78 | 154.00 | 344.00 | 65.60 | 15.50 | 11.00 | 29.80 | 739.00 | 725.00 | 250.00 | 152.00 | 3.47 | 4.72 | 8.74 | 5.32 |
|  | Tumorous-06 | 8150.00 | 1020.00 | 961.00 | 287.00 | 38.40 | 8.91 | 54.00 | 19.80 | 201.00 | 2010.00 | 501.00 | 70.50 | 38.30 | 82.90 | 2720.00 | 1690.00 | 562.00 | 392.00 | 13.10 | 24.30 | 41.60 | 33.90 |
| 7 | Non-tumorous-07 | 5220.00 | 733.00 | 740.00 | 83.10 | 60.40 | 8.12 | 48.60 | 22.90 | 158.00 | 1700.00 | 486.00 | 16.40 | 12.30 | 62.70 | 8760.00 | 907.00 | 434.00 | 196.00 | 39.90 | 13.90 | 11.80 | 10.20 |
|  | Tumorous-07 | 9060.00 | 809.00 | 944.00 | 226.00 | 46.40 | 4.84 | 33.10 | 10.20 | 151.00 | 1320.00 | 385.00 | 39.60 | 15.60 | 69.70 | 11400.00 | 1100.00 | 505.00 | 256.00 | 45.60 | 25.70 | 22.10 | 23.70 |
| 8 | Non-tumorous-08 | 6550.00 | 416.00 | 611.00 | 55.80 | 72.60 | 8.37 | 47.10 | 12.80 | 185.00 | 1100.00 | 58.40 | 10.50 | 7.40 | 195.00 | 9370.00 | 240.00 | 436.00 | 203.00 | 39.40 | 15.80 | 8.16 | 6.06 |
|  | Tumorous-08 | 8850.00 | 524.00 | 779.00 | 200.00 | 40.40 | 8.12 | 50.60 | 6.97 | 208.00 | 943.00 | 269.00 | 20.20 | 11.10 | 38.30 | 7260.00 | 325.00 | 445.00 | 97.70 | 24.60 | 26.60 | 20.30 | 19.50 |
| 9 | Non-tumorous-09 | 5490.00 | 391.00 | 530.00 | 98.30 | 49.50 | 6.47 | 43.20 | 11.60 | 250.00 | 1500.00 | 124.00 | 16.50 | 6.79 | 63.10 | 6840.00 | 182.00 | 376.00 | 76.40 | 38.50 | 15.90 | 9.78 | 6.55 |
|  | Tumorous-09 | 10000.00 | 444.00 | 927.00 | 108.00 | 41.90 | 5.77 | 32.20 | 3.68 | 174.00 | 958.00 | 63.00 | 20.80 | 9.95 | 19.60 | 9140.00 | 259.00 | 440.00 | 115.00 | 36.90 | 31.10 | 17.20 | 12.50 |
| 10 | Non-tumorous-10 | 7960.00 | 784.00 | 715.00 | 93.10 | 46.40 | 10.10 | 43.00 | 15.10 | 164.00 | 1200.00 | 148.00 | 30.70 | 15.30 | 49.60 | 9510.00 | 862.00 | 444.00 | 228.00 | 40.90 | 23.40 | 9.75 | 6.28 |
|  | Tumorous-10 | 5870.00 | 723.00 | 503.00 | 166.00 | 23.00 | 7.02 | 57.60 | 5.15 | 282.00 | 594.00 | 165.00 | 64.80 | 18.20 | 59.00 | 14600.00 | 757.00 | 492.00 | 159.00 | 61.50 | 18.80 | 27.60 | 13.60 |
| 11 | Non-tumorous-11 | 2510.00 | 196.00 | 194.00 | 39.30 | 34.60 | 3.16 | 18.80 | 21.60 | 153.00 | 405.00 | 180.00 | 9.93 | 10.80 | 46.00 | 1110.00 | 259.00 | 246.00 | 72.10 | 5.93 | 7.13 | 6.39 | 3.91 |
|  | Tumorous-11 | 11300.00 | 600.00 | 707.00 | 256.00 | 59.60 | 7.28 | 34.50 | 7.55 | 119.00 | 1600.00 | 254.00 | 11.70 | 31.60 | 48.10 | 3510.00 | 859.00 | 471.00 | 302.00 | 15.70 | 28.70 | 8.30 | 17.30 |
| 12 | Non-tumorous-12 | 1980.00 | 162.00 | 145.00 | 60.15 | 19.90 | 1.46 | 17.40 | 18.70 | 193.00 | 258.00 | 76.60 | 11.00 | 8.09 | 52.80 | 946.00 | 132.00 | 190.00 | 44.00 | 3.99 | 6.19 | 10.60 | 11.10 |
|  | Tumorous-12 | 4250.00 | 307.00 | 419.00 | 172.00 | 34.70 | 2.95 | 52.10 | 12.60 | 269.00 | 830.00 | 149.00 | 52.40 | 13.00 | 199.00 | 2720.00 | 209.00 | 422.00 | 130.00 | 11.20 | 14.80 | 34.50 | 15.10 |
| 13 | Non-tumorous-13 | 5090.00 | 1010.00 | 853.00 | 83.10 | 53.80 | 8.56 | 32.20 | 13.60 | 176.00 | 780.00 | 287.00 | 54.80 | 23.80 | 66.70 | 7930.00 | 940.00 | 618.00 | 152.00 | 46.90 | 17.70 | 8.80 | 16.00 |
|  | Tumorous-13 | 9410.00 | 608.06 | 837.00 | 150.00 | 43.40 | 6.39 | 80.40 | 15.90 | 174.00 | 1880.00 | 496.00 | 29.20 | 18.70 | 29.20 | 8010.00 | 1830.00 | 552.00 | 298.00 | 37.50 | 30.00 | 15.90 | 14.10 |
| 14 | Non-tumorous-14 | 2210.00 | 297.00 | 223.00 | 35.40 | 30.00 | 2.13 | 23.20 | 11.40 | 300.00 | 494.00 | 51.90 | 16.20 | 17.10 | 90.90 | 1360.00 | 369.00 | 236.00 | 249.00 | 7.30 | 8.14 | 10.50 | 6.43 |
|  | Tumorous-14 | 3990.00 | 364.00 | 354.00 | 29.10 | 19.90 | 5.46 | 31.70 | 5.33 | 241.00 | 563.00 | 81.90 | 19.60 | 8.04 | 10.60 | 4550.00 | 171.00 | 323.00 | 34.00 | 17.90 | 12.30 | 12.20 | 4.01 |
| 15 | Non-tumorous-15 | 2280.00 | 243.00 | 234.00 | 17.30 | 33.20 | 2.42 | 20.60 | 22.00 | 235.00 | 452.00 | 248.00 | 12.70 | 9.85 | 19.00 | 3990.00 | 289.00 | 251.00 | 83.30 | 25.50 | 7.66 | 5.12 | 2.55 |
|  | Tumorous-15 | 6240.00 | 822.00 | 639.00 | 55.60 | 34.40 | 10.00 | 41.10 | 12.00 | 279.00 | 1460.00 | 350.00 | 19.40 | 11.00 | 36.20 | 7020.00 | 983.00 | 344.00 | 227.00 | 37.80 | 21.60 | 14.20 | 5.79 |

Table S2. Detailed calculation process of Benjamini–Hochberg FDR correction for 23 statistical tests (22 elements and the Cu/Zn ratio).

|  | Rank (i) | Raw P  (uncorrected) | Criterion (i/m)*Q | Unadj. q (P*m/i) | Final q (BH) | Significant? |
| --- | --- | --- | --- | --- | --- | --- |
| P (μg/g) | 1 | 0.001 | 0.002 | 0.023 | 0.004 | Yes |
| Mg (μg/g) | 2 | 0.001 | 0.004 | 0.012 | 0.004 | Yes |
| Fe (μg/g) | 3 | 0.001 | 0.007 | 0.008 | 0.004 | Yes |
| Ti (ng/g) | 4 | 0.001 | 0.009 | 0.006 | 0.004 | Yes |
| Ga (ng/g) | 5 | 0.001 | 0.011 | 0.005 | 0.004 | Yes |
| Nb (ng/g) | 6 | 0.001 | 0.013 | 0.004 | 0.004 | Yes |
| Mo (ng/g) | 7 | 0.003 | 0.015 | 0.010 | 0.010 | Yes |
| Zr (ng/g) | 8 | 0.006 | 0.017 | 0.017 | 0.017 | Yes |
| Co (ng/g) | 9 | 0.007 | 0.020 | 0.018 | 0.018 | Yes |
| Sr (ng/g) | 10 | 0.011 | 0.022 | 0.025 | 0.025 | Yes |
| Ca (μg/g) | 11 | 0.025 | 0.024 | 0.052 | 0.048 | Yes |
| Rb (ng/g) | 12 | 0.025 | 0.026 | 0.048 | 0.048 | Yes |
| Cu/Zn ratio | 13 | 0.044 | 0.028 | 0.078 | 0.078 | No |
| V (ng/g) | 14 | 0.057 | 0.030 | 0.094 | 0.094 | No |
| Ba (ng/g) | 15 | 0.08 | 0.033 | 0.123 | 0.123 | No |
| Cs (ng/g) | 16 | 0.164 | 0.035 | 0.236 | 0.236 | No |
| Zn (μg/g) | 17 | 0.194 | 0.037 | 0.262 | 0.262 | No |
| Cr (ng/g) | 18 | 0.256 | 0.039 | 0.327 | 0.310 | No |
| Mn (ng/g) | 19 | 0.256 | 0.041 | 0.310 | 0.310 | No |
| Li (ng/g) | 20 | 0.293 | 0.043 | 0.337 | 0.337 | No |
| Ni (ng/g) | 21 | 0.336 | 0.046 | 0.368 | 0.368 | No |
| Cu (μg/g) | 22 | 0.507 | 0.048 | 0.530 | 0.530 | No |
| Pb (ng/g) | 23 | 0.944 | 0.050 | 0.944 | 0.944 | No |

Note: A total of 23 hypotheses were tested. At a false discovery rate (FDR) threshold of Q = 0.05, the largest significant rank was k = 12, corresponding to an unadjusted P-value cutoff of ≤ 0.026. Thus, the first 12 hypotheses were considered statistically significant after FDR correction.
